# Socio-Economic Factor Impact on the Republic of Buryatia (Russia) Green Economic Development Transition

**DOI:** 10.3390/ijerph182010984

**Published:** 2021-10-19

**Authors:** Alexey Bilgaev, Erzhena Sadykova, Fujia Li, Anna Mikheeva, Suocheng Dong

**Affiliations:** 1 Institute of Geographic Sciences and Natural Resources Research, Chinese Academy of Sciences, Beijing 100101, China; bilgaev@igsnrr.ac.cn; 2 Baikal Institute of Nature Management, Siberian Branch of the Russian Academy of Sciences, 670047 Ulan-Ude, Russia; ersadykova@binm.ru (E.S.); asmiheeva@binm.ru (A.M.); 3 Innovation Academy for Green Manufacture, Chinese Academy of Sciences, Beijing 100190, China

**Keywords:** Russia, Republic of Buryatia, Lake Baikal, green economy, composite indicator

## Abstract

Sustainable green development and environment preservation problems are relevant for unique territories with special economic activity modes, such as the Baikal natural territory. Within Russia, the Republic of Buryatia serves as the model territory for the Lake Baikal ecosystem preservation. Determining the socio-economic development impact on the region’s natural environment and resolving contradictions in transition to the green economic development requires the systematization of economic, social, and environmental processes of quantitative information based on the construction of composite indicators. We construct a composite indicator to assess the socio-economic factor’s impact on the Republic of Buryatia’s current economic state and to compare the current environmental subsystem state and the socio-economic parameters of Buryatia to the average Russian level. We use multiple regression models to determine relationships between various environmental-socio-economic parameters and identify the factors that most impact the environmental states (situations). The measures taken to preserve the unique ecosystem of Lake Baikal have an actual effect. This study shows, that according to the accepted scale, the environmental situation in the region can be characterized as a step towards the green economy transition. The proposed sustainable green development criteria and tools assessment system of the Republic may serve as the basis for forming information and analytical support for an effective economic policy.

## 1. Introduction

Environmental factors play a big role in the economic development of fragile nature regions with special nature management regimes and established restrictions on certain economic activities. The slowdown of economic growth has consequences for the well-being of the population. This situation leads to a conflict of interest between economic growth and environmental protection, as much of economic growth is still accompanied by environmental pollution, especially in developing countries [1]. In such regions, the current socio-economic development model must be changed to a more suitable green economy concept based on specific principles (internalize externalities; create decent work and green jobs; poverty reduction; protect biodiversity and ecosystems; promote resource and energy efficiency; use integrated decision making; have low carbon and low emissions; and maintain economic growth). These principles help to clarify the interpretation of the green economy concept by different international organizations and other stakeholders. They also help to guide practitioners in the application of the green economy concept and address perceived risks and concerns [2]. The term green economy was first coined during the late 1980s [3] in a pioneering 1989 report for the Government of the United Kingdom by Pearce et al. [4]—written by a group of leading environmental economists, it was titled “Blueprint for a Green Economy” [5]. However, during the 1990s and early 2000s the green economy concept seemed to have disappeared from common usage in international development circles [6]. This happened due to the advent of sustainable development, which captured political attention after the Rio Summit in 1992 [7]. In 2008, the term was revived in the context of discussions on the policy response to multiple global crises (financial crisis [8] and the environment problems that current socio-economic systems were encompassing [5,9] and sustainable economic growth [10]. The green economy concept is well established in the political sphere, and it appears in many policy agendas of international institutions and, currently, is more related to concepts linked to weak sustainability (i.e., energy efficiency or pollution control) [11]. It is perceived as a pathway to sustainability by international organizations such as the World Bank [12] and the United Nations Environment Programme [13]. There is no internationally agreed definition of green economy; UNEP has defined the green economy as “one that results in improved human well-being and social equity, while significantly reducing environmental risks and ecological scarcities. It is low carbon, resource efficient, and socially inclusive” [14]. In 2015, the UN developed and adopted the sustainable development agenda for a period until 2030, based on 17 sustainable development goals (SDGs) [15] with its own indicators framework [16,17]. Along with the rapidly expanding discourse about the transition to green economic development, scientists worldwide have begun to focus on ways to reduce the potential environmental impacts associated with human activities, including [18] emissions [19], lack of energy efficiency [20], transitioning to cleaner and cheaper energy [21], and renewable energy [22]), meanwhile, considering energy cost-efficiency [23], also taking into account economic risks [24]. Because sustainability activities are costly, this may become the case even more during times of financial constraint [25]. The modern assessment system in the economy in the light of the ongoing changes should be based on composite indicators—formed by a set of the most significant indicators interning into one—the need for integral assessment arises because particular indicators describing certain phenomena do not allow obtaining a comprehensive idea of the object of research. Another reason for conducting integral assessments is to understand the processes occurring with the object under study and their causes [26]. Composite indicators are synthetic indices or aggregates of all component indicators describing a multi-dimensional and often complex issue [27]. Composite indicators and ranking systems aggregate multi-dimensional processes into simplified concepts that are often used for advocacy and policy consumption [28]. They have been used in many contexts, from economics, through engineering and planning, to environmental sciences [29].

However, it remains unclear how the region will achieve sustainable development goals and how it will affect economic development. Therefore, it is not enough to study the general environmental state indicators of the region. It is necessary to conduct a comprehensive assessment of its environmental-socio-economic development. Despite the growing amount of studies focused on the green economic development of the Republic of Buryatia, the Baikal region, and environmental and economic interactions at the regional level, the problem of finding ways of sustainable development to preserve unique natural objects, which include Lake Baikal, is still not studied enough. Most of the existing research focuses on theoretical approaches to the transition to a green economy in the region and an empirical analysis of the environmental situation [30,31,32].

This study aims to comprehensively assess the impact of the socio-economic factor on the transition of the Republic of Buryatia to green economic development. The research is carried out in the Republic of Buryatia [33]—the Baikal natural territory (BNT), with an established special regime of nature management (Federal Law “On the Protection of Lake Baikal” dated 01.05.1999 N 94-FZ). For the first time, we develop a methodology to assess the environmental-socio-economic development of the Republic of Buryatia under the impact of current economic conditions by constructing a composite indicator based on three groups of indicators—economic, social, and environmental. We propose a system of criteria-based assessments of the composite indicator—an essential toolkit for decision-makers in state regulation of the achievement of green economic development—to measure its progress. Regional environmental-socio-economic system has a potential for dynamic development. The study showed that the composite indicator is a parameter that any region within its boundaries, including material, social, and natural flows, can function and develop sustainably. Therefore, this study might be helpful when studying similar regions.

## 2. Materials and Methods

### 2.1. Study Area

The Republic of Buryatia is a federal subject of the Russian Federation, with its capital in the city of Ulan-Ude. The area is 351,334 km² (2.05% of the territory of Russia). It has a population of 985 thousand people. The Republic is located in the center of Asia, in the south of Eastern Siberia. The large territory stretching from the south-west to the north-east determines the different conditions of economic management and stay in the region. The relief is characterized by mountain ranges with vast, deep, and almost closed intermontane basins. The climate of Buryatia is sharply continental. It formed under three contrasting components: the dry and cold climate of the northern regions, the hot and dry Mongolian deserts, and the humid Pacific. Winter is cold with dry frost, and the main snowfalls fall in November-December. Spring is windy, with prevailing north-westerly winds, frost, and almost no precipitation. It has a short summer, with hot days and cool nights with heavy rainfall in July–August; autumn comes without a sharp change in weather. An essential feature of the climate of Buryatia is the long duration of sunshine—1900–2200 h, which is not less than in the southern regions of Russia. Buryatia river flow resources are 98 km³; there are 94.3 thousand m³/year per inhabitant (almost three times more than the average in Russia); and 279.8 thousand m³/year per 1 km² of territory. A total of 61% of the river flow of the Republic falls in the basin of Lake Baikal.

Lake Baikal and the Baikal natural territory have a special status, enshrined not only at the federal but also at the world level as a UNESCO World Heritage Site (1996). The Baikal region covers the southeast of Siberia and the northern part of Mongolia, with a total area of more than 1 million km^2^. The core of the Baikal region is the Baikal natural territory. The area of the water surface of Lake Baikal is 31,500 km^2^.

**Figure 1:**
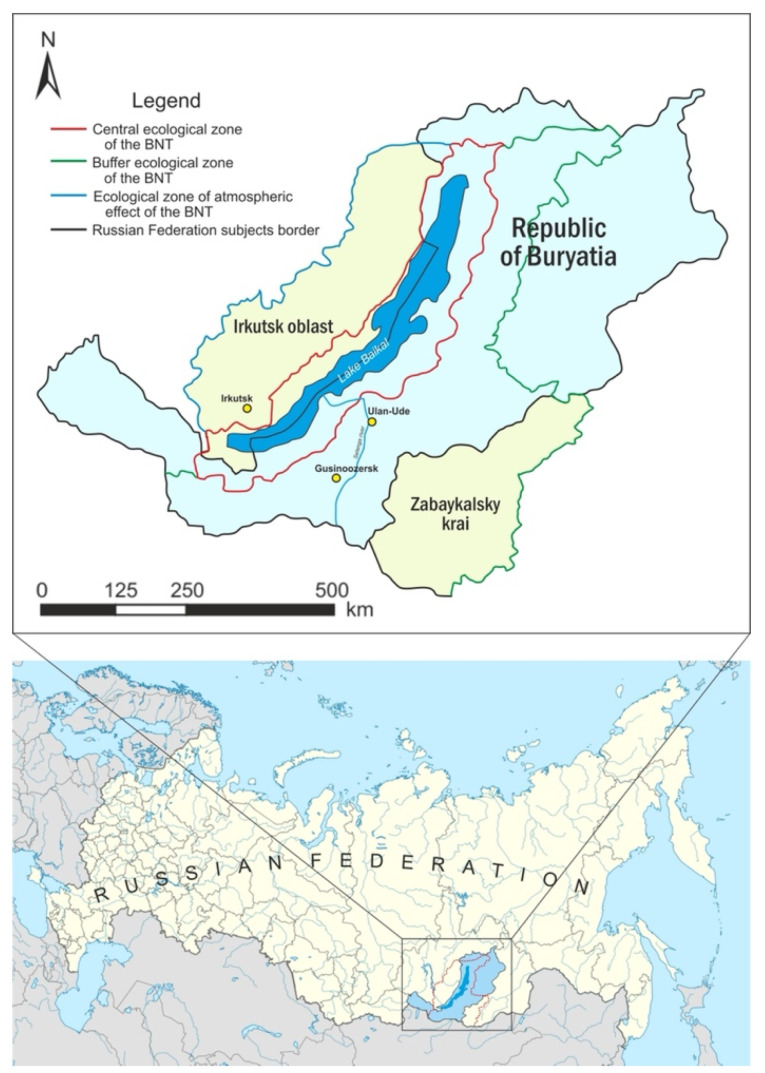
Baikal natural territory (BNT).

The BNT within Russia covers three regions: the Republic of Buryatia (73%), Zabaykalsky Krai (21%), and Irkutsk Oblast (6%); this includes Lake Baikal, a water protection zone adjacent to Lake Baikal. Its catchment area is within the Russian Federation, special protected natural areas adjacent to Lake Baikal, as well as in an area up to 200 km wide to the west adjacent to Lake Baikal, and north-west of it (Figure 1).

There are three ecological zones:

- Central zone—a territory that includes Lake Baikal with islands, a water protection zone adjacent to Lake Baikal, as well as specially protected natural areas adjacent to Lake Baikal;
- Buffer zone—the territory outside the central environmental zone, including the catchment area of Lake Baikal within the territory of the Russian Federation;
- Atmospheric influence zone—an area outside the catchment area of Lake Baikal within the territory of the Russian Federation, up to 200 km wide to the west and north-west of it. This is the location of economic facilities with activities that harm the unique ecosystem of Lake Baikal. The Government of the Russian Federation regulates the ecological zoning of the Baikal natural territory.

The established special regime of economic activities imposes on the Republic of Buryatia the most significant responsibility for Lake Baikal purity. To protect the unique ecosystem of Lake Baikal, a special regime of economic activities is carried out by the principles of priority actions that do not violate the ecosystem of Lake Baikal and its natural landscape protection zones, considering the complexity of economic activity impacts on the Lake Baikal ecosystem. This includes the sustainable development-based solutions of socio-economic and Lake Baikal protection tasks and mandatory state environmental expertise. Environmental protection becomes an essential requirement for the development of any production under the conditions of “greening the economy”; the effective use and reproduction of natural resources in the Baikal region, where industrial production is the leading industry, and the presence of Lake Baikal determines the promising directions of future economic activity.

### 2.2. Research Methodology

The developed conceptual provisions are based on the following research procedure.

1. Analysis of the intensity of structural shifts in the economy of the Republic of Buryatia and assessment of production diversification.
2. The Republic of Buryatia’s environmental state in terms of the main types of impact on the environment and determination of the industry contribution to pollution.
3. Analysis of the Republic of Buryatia’s current trends in the environmental sphere using the relative specific indicators eco-capacity and eco-intensity, which characterize the level of environmental pollution.
4. The developed toolkit to assess the socio-economic factor impact on the transition to green economic development is based on statistical analysis methods.
5. The assessment of factors affecting the environmental situation in the Republic of Buryatia made it possible to determine the composition of particular indicators included in the calculation of the composite indicator.
6. The developed diagnostics and assessment methodology, based on the calculation of particular and composite indicators; the proposed system of criteria-based evaluations of the composite indicator and the obtained indicators measurement scale made it possible to determine steps on the way to sustainable green economic development.

### 2.3. Statistical Methods

The method of constructing time series and the method of structural shifts allowed us to conduct a comparative analysis of indicators that characterize the environmental and socio-economic development of the Republic of Buryatia. In this study, a correlation and regression analysis of main indicators of the Republic of Buryatia was carried out in order to identify the factors that have the greatest impact on the Republic of Buryatia’s environmental state and the relationship between various components of the economy and the environment. To solve this problem, we used a Statistica 8.0 (StatSoft, Palo Alto, CA, USA) application.

To identify and assess the factors affecting the environmental development of the Republic of Buryatia, a model of the multiple regression equation, described by a function [34]:

(1) $${\bar{Y}}_{1,2,\ldots ,k}=\;f\left(x_{1},{\;x}_{2},\;\ldots ,{\;x}_{k}\right),$$

where ${\bar{Y}}_{1,2\ldots ,k}$—regressand; x—regressor; f(x)—some function, according to it, where goes the interaction of the variables Y and x.

To build a multivariate relationship model, we used a linear multiple regression model:

(2) $${\bar{Y}}_{x}={\;a}_{0}+a_{1}x_{1}+a_{2}x_{2}+a_{3}x_{3}$$

The conducted statistical analysis of target indicators using the Statistica 8.0 software made it possible to identify the main factors influencing the dynamics of the Republic of Buryatia’s environmental-socio-economic state. The use of multiple regression equations made it possible to analyze factor signs and their selection for a composite indicator.

### 2.4. Composite Indicator Calculation Method

The developed toolkit for a comprehensive assessment of the region’s environmental-socio-economic development is based on the method of multivariate comparative analysis, index method, method of normalization and standardization, and cumulative (summation) method.

To normalize the indicators, we took the corresponding indicator values of the federation subjects as the reference points. From the adopted indicators characterizing environmental-socio-economic state of the federal subjects, we selected corresponding indicators with the values *x_min_* and *x_max_* in 2018. We took statistical data of the Republic of Buryatia for 2018 as the fact values of the indicators *x_fact_*. The indicators were standardized using the linear scaling method, which allows us to track the dynamics of each indicator’s actual growth/decline relative to stable reference points (maximum and minimum values of the indicator).

Linear scaling formula:

(3) $$x_{st}=\frac{x_{fact}-x_{min}}{x_{max}-x_{min}}$$

Standardization of (*fact*) values using threshold estimated intervals of individual indicators made it possible to move to a single dimensionless value to calculate aggregate indicators for the environmental-socio-economic components of a region and then calculate a composite indicator.

At the same time, the region’s environmental-socio-economic development level was measured in a range from 0 to 1, with the fixation of the possible limits of the boundaries for positioning the research object in the external and internal environments. The weighted coefficients of the indicators (*f_i_*) were taken as equal weights of the entry into the formula.

Legend for calculating the region sustainable development composite indicator:

(4) $$I_{j}=\overset{n}{\underset{i=1}{\sum }}x_{ist}^{j\;}\times f_{i}^{j}$$

(5) $$I_{reg}=\sqrt[{3}]{I_{econ}\times I_{soc}\times I_{env}}$$

$I_{j}$—aggregated indicator of the *j*-th component of the region’s economy;

$x_{ist}^{j\;}$—standardized value of the *i*-th indicator for the *j*-th component of the regional economy;

$f_{i}^{j}$—weighted coefficient of the *i*-th indicator of the *j*-th regional economic system;

$I_{econ}$—aggregated indicator of the region’s economic development;

$I_{soc}$—aggregated indicator of the region’s social development;

$I_{env}$—aggregated indicator of the region’s environmental development.

$I_{reg}$—composite indicator of the region’s sustainable development.

To monitor individual stages on the path to achieving (deviations in the negative or positive direction) development, we developed the composite and aggregated indicators assessment scale for the boundaries of the threshold values (Table 1). This made it possible to determine the step-by-step stages of the regional economy transition, depending on its development type, and to interpret the obtained data. Achieving the conditions of the metaphase in the transition from one type of development to the next ascending allowed us to establish the certain existing opportunities at each stage, with their subsequent adjustment to accelerate the transition to the green economic development.

The proposed system of criteria and tools in assessing region development may serve as the basis for forming information and analytical support for an effective environmental policy. At the same time, we should emphasize that the specific composition of indicators may vary depending on the goals and objectives at each stage of the region’s development. The proposed approach can be concretized and interpreted according to the existing objective distinctive features of individual regions.

## 3. Results

### 3.1. Sectoral Structure of the Economy of the Republic of Buryatia

**Figure 2:**
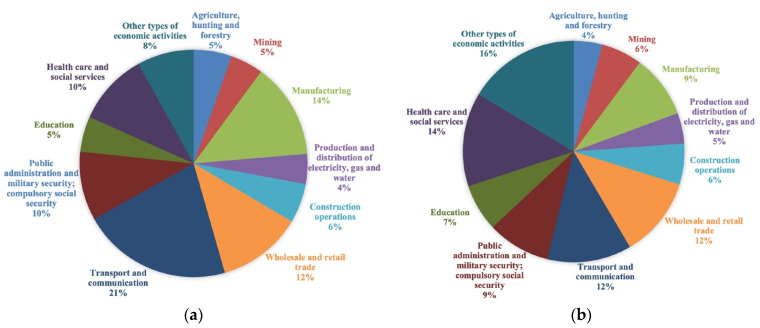
Structure of Republic of Buryatia’s economy (**a**) 2010; (**b**) 2018.

At present, the Republic of Buryatia is an industrial-agrarian republic. It is a large region of mining, forestry, and mechanical engineering. Against the background of the Russian Federation, it stands out for reserves of rare and non-ferrous metals, gold mining, and developed fur trade. In recent years, in terms of socio-economic development, Buryatia has taken an intermediate place between the middle and weakest regions of Russia. Let us consider the changes in the structure of the gross regional product of the Republic of Buryatia for the analyzed period. Figure 2 shows the sectoral structure of GRP by type of economic activity in 2010 and 2018.

The economic structure should ensure the harmonious and environmentally acceptable use of natural and economic conditions for a certain period following the set goals. Therefore, when studying the structure of the economy, from our point of view, the central place should be given to identifying trends in structural shifts from the standpoint of compliance or their approximation to the main social and environmental imperatives; this is especially true for areas with environmental restrictions as in the Republic of Buryatia.

The analysis showed that the most dramatic structural shifts were observed in the following industries: the share of agriculture, fishing, and fish farming decreased by 21.05%, processing industries—30.77%, transport, and communications—41.07%. Accordingly, the share of the raw materials sector increased by 30.61%. Relatively favorable trends include an increase in the share of education—39.62% and in health care and provision of social services—36.11%. Structural shifts in the Republic’s economy were mainly negative. Production diversification has decreased in the industry structure; the most significant share began to be occupied by the electric power industry and non-ferrous metallurgy due to influence of the price factor. In recent years, in the Republic of Buryatia, as in most Russian regions, the sectoral structure has become less progressive, reminiscent of developing countries’ structure with a raw material export orientation.

### 3.2. Environmental Pollution by Type of Economic Activity

**Figure 3:**
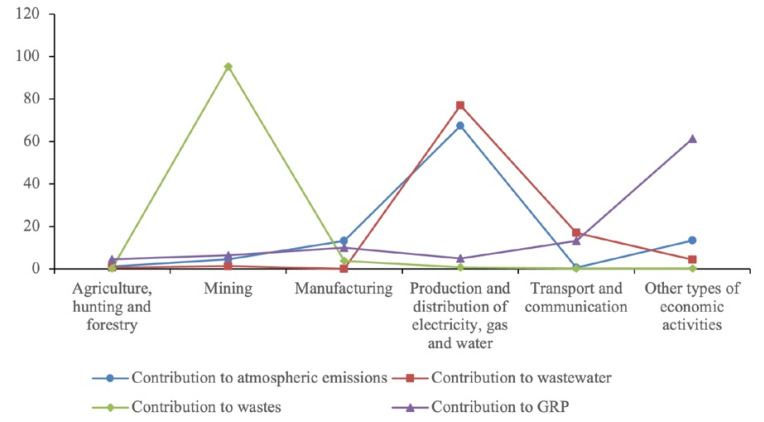
Industry contribution into pollution and GRP 2018, in percentages.

Assessment of the environmental state and well-being of natural systems, their adequate protection, and rational use in sustainable and green development are among the most important international, national, and regional problems. Generally, the assessment of the environmental state of the region is carried out according to the following main types of impact on the environment—pollution of water resources; air pollution due to emissions of pollutants into the atmosphere from stationary and mobile sources; soil pollution due to waste disposal. To measure the economic activity efficiency ratio with the level of its impact on the environment, we determine how high the intensity of the main pollution would be by type of production and leading industries of the Republic, as well as how ambiguous the contribution of operating enterprises would be to the existing scale of pollution in the environment. We carried out calculations and compared the volumes of environmental pollution by industry with their respective contributions to GRP (Figure 3).

The graph shows the share of sectors of the economy with the most significant volumes of pollution by main types: air pollution, wastewater discharge, and waste disposal, compared with the share of these sectors in the GRP. In terms of the most significant share of pollutant emissions into the atmosphere—67.4%, the first place is occupied by the production of electricity and water (stationary sources). Also, in recent years, pollution emissions from vehicles have sharply increased. The second group of industries is the manufacturing sector, accounting for 13.2% of total air pollution. For the Republic of Buryatia, indicators of wastewater discharge and their pollution with harmful and hazardous substances are very important. The electric power industry is the leader in polluted water discharge—76.8%. Accordingly, the industry’s share in the GRP is 4.8%. The next type of pollution that poses a severe environmental hazard is production and consumption waste. The formation of the bulk of waste observed in mining is accompanied by a large volume of overburden; therefore, in the total volume of waste, this type of economic activity accounts for 95.3%. Accordingly, the contribution of this industry to the GRP is 6.4%.

### 3.3. Eco-Capacity and Eco-Intensity Indicators

The stability of the regional natural system is determined by the need for timely adoption of measures that are adequate for the environmental threat of negative consequences from air pollution, discharge of polluted wastewater, and accumulated waste. We used the relative specific indicators, eco-capacity and eco-intensity of environmental pollution, to characterize the current trends in the environmental sphere of the Republic of Buryatia. Table 2 compares eco-capacity and eco-intensity of emissions, discharges of pollutants, and production and consumption waste in the Republic of Buryatia for 2010–2018.

Eco-capacity shows the specific load of the negative impact of the produced pollution on the person. The data in the table indicate a positive trend in Buryatia, indicating a tendency towards a decrease in eco-capacity and eco-intensity in terms of air and water pollution. At the same time, the tensest situation is waste. Therefore, for the period of 2010-2018, eco-capacity of pollutant emissions decreased by 4.5%, including emissions from stationary sources that decreased by 2.31%; the Republic’s population increased by 1.4%; and 87% of the pollutants supplied to the treatment plant are captured and neutralized, influencing the decrease in the eco-capacity of air. Also, positive dynamics were noted in eco-capacity of water resources and the overall decrease was 18.6%. The overall decrease in the discharge of polluted wastewater was of 17.4% and influenced the decrease in the eco-capacity of the water. We observed the opposite with waste disposal. Eco-capacity of waste for the analyzed period increased by 4.75 times. The main unfavorable factor is a sharp increase in waste generation in the mining sector of the economy.

The analysis of eco-intensity indicators showed negative changes aimed at reducing air pollution and discharges per 1 USD (GRP), which amounted to 19.5% and 0.9%, respectively. As noted above, high production and consumption waste generation rates are due to an increase in mining volume. Waste disposal is one of the essential tasks in order to prevent the negative impact on the environment of the accumulated amount of harmful substances in the past. Of the total volume of waste generated in 2018, 25,867 thousand tons (32.1%) were used and neutralized.

For a complete description of the current situation in the Republic of Buryatia environmental sphere, a comparative analysis of the dynamics of the Russian Federation and Buryatia GRP growth rates and a comparison of the eco-capacity indicators growth rates deserves attention. Figure 3 shows the comparative dynamics of the indicators of the Russian Federation and the Republic of Buryatia.

**Figure 4:**
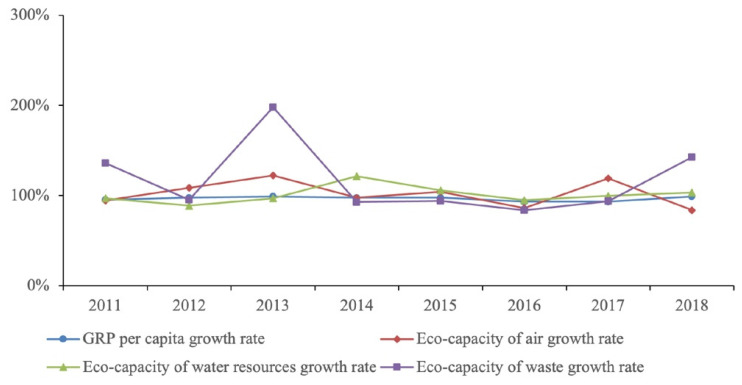
GRP per capita growth rate compared to eco-capacity growth rate in 2010–2018 in RB and Russia (2010 = 100%).

Regarding the GRP per capita growth rate, the Republic of Buryatia lags behind the Russian Federation by an average of 3.3% (Figure 4). According to this indicator, Buryatia occupies the 75th position out of 85 federal subjects of Russia. In terms of the eco-capacity of atmospheric air, water resources, and waste, over the past period, there was on average an excess of the rate of decline of these indicators in the Republic of Buryatia, compared to the Russian Federation, i.e., the intensity of measures taken in Buryatia to reduce anthropogenic pressures on the environment is insufficient.

### 3.4. Factors Influencing the Republic Buryatia Environmental Situation

We assessed factors influencing the environmental situation of the Republic of Buryatia. The results of the correlation analysis for the Republic of Buryatia provided below reflect the resulting indicators: multiple regression equations, factors affecting the indicator under consideration, F-test F value, and F critical value, which was used to check the quality of the regression equation. Based on the constructed multiple regression equations, the results obtained indicate that the following factors had the greatest impact on the environmental development of the Republic of Buryatia. In general, in the Republic of Buryatia, the main resulting indicators were most influenced by eight factors:

1. Gross regional product (GRP) per capita, USD;
2. Per capita income of the population, USD
3. Eco-intensity of air, kg/USD;
4. Industrial production per capita, USD
5. Population with income below the subsistence minimum, %.
6. Fixed investment per capita, USD;
7. Employment rate, %;
8. Unemployment rate, %

Results of the correlation analysis for the Republic of Buryatia.

1. Eco-capacity of air pollution
  $$Y=79.46858-0.00966x_{1}+0.10827x_{2}$$
  *x*_1_—Gross regional product (GRP) per capita, USD;
  *x*_2_—Per capita income of the population, USD
  F value (6.13) = 4.7787 *p* < 0.00876; F critical value = 7.86; F value > F critical value.
2. Eco-capacity of pollution of water resources
  $$Y=-790.116+14.717x_{1}-0.077x_{2}+3.665x_{3}$$
  *x*_1_—Eco-intensity of air pollution, t/USD;
  *x*_2_—Industrial production per capita, USD
  *x_3_*—Population with income below the subsistence minimum, %
  F value (15.14) = 12.643 *p* < 0.01243; F critical value = 5.85; F value > F critical value.
3. Eco-capacity of waste
  $$Y=-410.1248-0.3555x_{1}-8.2835x_{2}-5.3688x_{3}\;$$
  *x*_1_—Fixed investment per capita, USD;
  *x*_2_—Employment rate, %;
  *x_3_*—Unemployment rate, %
  F value (10.9) = 10.572 *p* < 0.00077; F critical value = 9.89; F value > F critical value.
4. Eco-intensity of air pollution
  $$Y=-1.25259+0.00475x_{1}+0.58664x_{2}+0.00932x_{3}\;$$
  *x*_1_—Industrial production per capita, USD;
  *x*_2_—Unemployment rate, %;
  *x_3_*—Per capita income of the population, USD
  F value (9.10) = 19.507 *p* < 0.00003; F critical value = 9.2; F value > F critical value.
5. Eco-intensity of pollution of water resources
  $$Y=-805.59-32.74x_{1}+4.1x_{2}\;$$
  *x*_1_—Per capita income of the population, USD;
  *x*_2_—Gross regional product (GRP) per capita, USD
  F value (8.11) = 20.276 *p* < 0.00002; F critical value = 8.66; F value > F critical value
6. Eco-intensity of waste
  $$Y=9222.494-0.578x_{1}-182.312x_{2}-78.327x_{3}\;$$
  *x*_1_—Fixed investment per capita, USD;
  *x*_2_—Employment rate, %;
  *x_3_*—Unemployment rate, %.
  F value (10.9) = 12.878 *p* < 0.00035; F critical value = 8.96; F value > F critical value

Based on the identified interrelationships of factorial features, we determined the main indicators included in the calculated data for constructing the composite indicator.

### 3.5. Composite Indicator

As a result of the correlation–regression analysis, we adopted a system of indicators based on the assessment of factors affecting the environmental development of the Republic of Buryatia (Table 3).

Standardization of actual values using threshold estimated intervals of individual indicators made it possible to move to a single dimensionless value for calculating aggregated indicators of the Republic of Buryatia’s economic, social, and environmental development. Table 4 shows the calculated aggregated indicators and the composite indicator for the analyzed period.

According to the accepted scale, economic development in Buryatia from 2010–2011 characterizes as medium-low; from 2012 to 2018, the obtained values of the composite indicator of economic development showed a weak level of economic development. The dynamics of the consolidated social indicator for the analyzed period in 2013 reached a value of over 0.5, but by 2018 it fell to 0.3988, repeating the negative trend in economic development; on the scale, this is a medium-low level. According to the composite indicator, the assessment of the environmental situation showed that the environmental development had a medium-high level.

**Figure 5:**
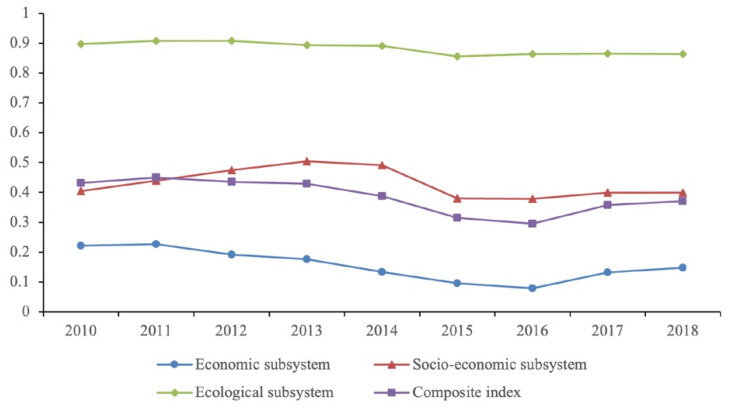
Dynamics of summary and composite indicators of the Republic of Buryatia for 2010–2018.

Figure 5 shows the influence of socio-economic factors on the environmental situation in the Republic of Buryatia.

Assessment of the development of the Republic of Buryatia using the composite indicator showed that the dynamics of the aggregate assessment indicators makes it possible to trace the trajectory of the main trends in the development of the republic. According to the accepted scale of measurements, the type of medium-low development was typical for the Republic of Buryatia for the analyzed period. The obtained results were influenced by the Russian Federation’s economic development slowdown, caused by the economic crisis of 2014–2015 and reflected in the dynamics of the main macroeconomic indicators of the Republic of Buryatia. It is reflected in the indicators of social development. In general, the Republic lagged behind the average Russian level in a number of socio-economic indicators.

## 4. Discussion

1. The analysis of environmental pollution by type of economic activity in the Republic of Buryatia shows that the most environmentally hazardous industry responsible for the pollution of atmospheric air and water resources is the power industry. The main reason for the high anthropogenic load on the environment is the use of cheaper, low-quality coal on farms and energy enterprises; the second reason is the increase in freight, passenger, and personal vehicles. The Lake Baikal basin special management regime imposed higher environmental requirements on electric power facilities; therefore, the main environmental measures should be carried out, first of all, in this industry. It should be noted that Buryatia has a significant amount of energy resources for the development of renewable energy. The peculiarities of climatic conditions include a long duration of sunshine and strong winds, making it possible to use these available sources. It is necessary to replace coal-based electricity generation with sustainable resources. China is one example of the construction of wind farms. In 2020, China built 71.67 GW of new wind farms, according to the country’s National Energy Administration (NEA). This is an absolute annual record—the commissioning of new wind farms in the country exceeded by almost three times the indicator of 2019. Moreover, it turns out that the capacities of wind farms commissioned in China over the past year exceed the total capacities of all new similar power plants worldwide in 2019 60.4 GW) [35]. Thus, the three rapidly growing renewable energy sectors (solar hot water, solar photovoltaic, and wind power) are significant contributors to green economies in China [36]. This trend started from China’s 11th Five Years Plan when China prioritized green development in almost all of its leading economic sectors [37]. According to Bloomberg NEF experts, at the moment, wind energy is winning—it generates two times more electricity due to the higher utilization of installed capacity [38]. In the coming years, the Republic of Buryatia will complete two solar power plants (SPP) in the Dzhidinskiy district of Buryatia. By 2022, seven SPPs will operate in the Republic in 6 out of 23 districts, with 145 MW capacity in total [39]. In order to reduce anthropogenic pressure on water resources, it is necessary to introduce more modern and technologically complex solutions, starting with the construction of treatment facilities using innovative solutions. This requires coordination and consideration of the interests of all of the subjects of water use, the adoption of preventive measures, and the improvement of the economic mechanism of water use. To solve the waste neutralization problem, it is necessary to create a database on waste and processing methods, introduce a monitoring system, and waste use economic incentives.

2. Analysis of the relationship between various components of the economy, social sphere, and environment showed quantitative characteristics of the influence, economic, and social factors on the resulting indicators characterizing the environmental situation.

The industrial–production potential of the Buryatia for the analyzed period decreased; there was deformity of the sectoral and spatial structures of the economy due to increased raw material orientation. Increased internal differentiation of population incomes has changed the organizational and institutional conditions in the development industry and other sectors of the economy. Despite the measures taken to protect Lake Baikal, we note that the environmental situation in the Republic remains difficult. Although there has been a lag in the rate of anthropogenic load reduction on all components of the environment from the dynamics of production volumes, the level of environmental pollution remains high and threatens the unique ecosystem of Baikal.

The social consequences include the constant outflow of the population from Buryatia, especially young people. The main reason is a significant lag in the population’s quality of life from the more developed regions of Russia. For example, the average per capita income of the Republic population is 1.4 times lower than the national average and the unemployment rate remains high (9.3%). There are a number of problems in the sphere of employment in the Republic, which are: first, deformation of the structure of the economy; secondly, the existing imbalance in the demand and supply of labor; third, ineffective employment (the predominance of low-paid jobs, an excess of unprofitable enterprises, and insufficient development of small businesses); and fourth, the expansion of the scale of informal employment. To overcome the existing backlog and rectify the situation that has developed in the regional labor market, it is necessary to take the following measures: to provide freely chosen employment, to create conditions for choosing a specialty in accordance with the professional capabilities of each person, in order to strengthen the motivation for effective work.

3. The assessment of the impact of economic conditions on the environmental situation by constructing a composite indicator of economic, social, and environmental development made it possible to identify the prevailing trends in the Republic’s development. The proposed method of indicator systems can be adopted as the assessment tool to stabilize the environmental situation, and the adopted scale of the threshold values of the composite indicator will determine the overall average level of the environmental-socio-economic development of the region.

Calculated values of the composite indicator of environmental development obtained have shown that the measures taken to preserve the unique ecosystem of Lake Baikal have an actual effect. According to the accepted scale, the environmental situation in the region can be characterized as a step towards the green economy transition. The contradictory nature of the situation lies in the fact that the environmental restrictions imposed on the production and economic activities of industrial, agricultural, and other enterprises of the Republic of Buryatia have generally positively influenced the environmental situation. On the other hand, the assessment of the economic factor indicates that there are certain difficulties in the Republic, due to the established economic regime. The resolution of the existing contradictions is now possible only with the intensification of actions on state structural bodies.

In current conditions, the transition to sustainable green economic development should be based on restructuring the economy (in the national economy sectors that harm the environment, employment, living conditions, and reduce the outflow of population from rural areas) and strengthening resource and energy conservation policy. It is especially relevant for territories with strict regulations in natural resource use and the general economic activities to which the Republic of Buryatia belongs. Therefore, for Buryatia, the essence of an environmentally balanced transformation of the economy’s structure is to stabilize production growth, primarily in the extractive industries, and the fuel and energy sectors within the Baikal natural territory. This can be done subject to the rapid development of modern technologies in the use and processing of mineral raw materials, which will require a redistribution of resources in favor of environmental-technological types of production. Also, it is necessary to create and modernize transport and energy infrastructure in order to create new green jobs [40]; and develop eco-tourism that does not violate the ecosystem’s integrity and is beneficial to the local population [41].

4. There are economic risks on the way to achieving green development in the region. The transformation of the economy’s structure and the transition to an environmentally sustainable green type of management will increase production costs. It will be necessary to re-equip the leading enterprises of the fuel and energy, machine-building, forestry, and agro-industrial complexes with resource-saving and environmentally friendly technologies. Within the central ecological zone directly adjacent to Lake Baikal, which has the strictest regulation of economic activities, it is possible to develop hunting, sport, and green tourism. The Republic, which is the center of Buddhism in Russia, has a majestic and untouched nature; the landscape features of the coast, the unique beauty of Lake Baikal, and the ethnocultural characteristics of the peoples living on the territory of the Republic, determine the high competitiveness of such types of tourism. Development of infrastructure are based on the widespread use of recreational and balneological resources and resorts; the development of small businesses in gathering and processing wild plants will increase employment and incomes of the population. The solution to the problem of leveling inequality in the conditions of economic activity is possible with government support and the development of a mechanism for compensating production costs due to high environmental requirements. For this, it is necessary to provide a legal framework for adopting regulations by the Russian government on compensation for the additional environmental costs of producers of the republican budget.

5. We propose implementing a model project to transition from a raw material orientation to an innovative path in the Baikal region (BNT regions) economy, where the incomes of competing regions will depend on organizational and technological innovations. Within the framework of a single state in neighboring regions with economically unbalanced systems, there is an obvious need to form integration processes for effective interaction in developing a macroregion based on a green economy. We suggest establishing a special interregional Baikal Region Green Development Fund and launch of the Baikal Region Green Development State Program. The regional governments will perform the regulatory function within the Baikal region [1]. First of all, this should determine the forms and methods of state support; determine the list of organizations entitled to subsidies and subsidies; and establish the procedure for granting subsidies and reporting. The main idea is to restructure the incoming public funds; the essence is to redistribute financial flows from polluting brown industries to green ones [42]. When introducing a mechanism for interregional environmental and economic integration, it is necessary to take into account the interests of business owners—it is required to create regulatory legal acts that will minimize the potential decline in the profitability of energy and other businesses, which arose through territorial preferences—to smooth out the greening of industries as much as possible.

It is necessary to provide enterprises with subsidies for introducing green technologies and industries, give grants for green developments, and partially reimburse the costs of re-equipment and the introduction of green technologies into existing production; subsidized rates, credits, and tax credits. It needs to provide the enterprises with staff—the organization of training courses and retraining courses. It is necessary to involve scientific institutes to analyze and forecast the processes of environmental-socio-economic processes; this will allow one to quickly and accurately understand the current situation and work out development strategies for each enterprise and to attract private companies to participate in green projects [43].

The proposed system of criteria and tools in assessing the sustainable green development of the Republic will serve as the basis for the formation of information and analytical support for an effective economic policy. Thus, the sustainable green development of the Republic of Buryatia should become a real solution to socio-economic problems for the formation of a better economic model and greening the economy.

## 5. Conclusions

Assessment of the socio-economic factor impact on the Republic of Buryatia environment using the composite indicator showed the relationship between regional subsystems and the main trends in the Republic development. The environmental situation can be characterized as a step towards the transition to a green economy. However, there are certain difficulties in the region due to the current economic regime. Therefore, the transition to sustainable “green” economic development should be carried out based on economic restructuring, which is possible only with the intensification of government action.

To implement a model project for the transition from a raw material orientation to an innovative path in the economy of the Baikal region (BNT regions), we propose establishing the Baikal Region Green Development Fund, which will allow launching the “Baikal Region Green Development State Program”. At the same time, it is necessary to determine the forms and methods of state support. The introduction of the mechanism of interregional environmental and economic integration will allow taking into account the interests of business owners. Furthermore, providing subsidies to enterprises for introducing green technologies, grants for green development, and partial reimbursement of the costs of introducing green technologies, will support a quick and accurate understanding of the current situation.

The proposed system of criteria and tools in assessing the green development of Buryatia is an essential toolkit for decision-makers in state regulation of the achievement of green economic development to measure its progress. Moreover, it may serve as the basis for forming info-analytical support for an effective economic policy. The green development of the Republic of Buryatia will solve socio-economic problems and green the economy.

## Figures and Tables

**Table 1:** Regional economic development aggregated and composite indicators measurement scale.

Development Type	Boundaries
High	1.0–0.9
Medium–high	0.8–0.7
Medium	0.6–0.5
Medium–low	0.4–0.3
Low	0.2–0.1

**Table 2:** Indicators of eco-capacity and eco-intensity of the Republic of Buryatia in 2010–2018.

2010	2011	2012	2013	2014	2015	2016	2017	2018	2018 to 2010	2018 to 2017
**Eco-capacity of air (kg per capita)**										
98.0	93.0	103.0	118.0	108.0	111.0	95.5	114.8	94.5	96.5	82.3
**Eco-capacity of water resources (m^3^ per capita)**										
43.7	40.9	35.6	33.4	38.8	40.0	38.6	35.6	35.6	81.4	100.0
**Eco-capacity of waste (t per capita)**										
17.23	26.96	29.86	60.72	55.66	51.23	45.97	49.17	81.82	474.8	166.4
**Eco-intensity of air (kg/USD)**										
0.022	0.017	0.019	0.021	0.020	0.034	0.031	0.033	0.026	119.5	79.4
**Eco-intensity of water resources (m^3^/USD)**										
0.010	0.007	0.007	0.006	0.007	0.012	0.012	0.010	0.010	100.9	96.5
**Eco-intensity of waste (kg/USD)**										
3.818	4.946	5.484	10.737	10.486	15.490	14.832	14.002	22.470	588.5	160.5

**Table 3:** Indicators for calculating the composite indicator.

Subsystems of the Regional System		Indicators
Economic subsystem	1	GRP per capita, USD
	2	Fixed investment, % to GRP
	3	Fixed investment per capita, USD
	4	Fixed investment, aimed at environment protection, % to GRP
Subsystem of social development	1	Per capita income, USD
	2	Ratio of the average wages of the regions with their average size in Russia, %
	3	Population with income below the subsistence minimum, % to the total population of the region
	4	Unemployment rate, %
	5	Gini coefficient
Environmental subsystem	1	Economy main sectors contribution to air pollution, %
	2	Economy main sectors contribution to water pollution, %
	3	Economy main sectors contribution to waste generation, %
	4	Eco-intensity of air kg/USD
	5	Eco-intensity of water resources m^3^/USD
	6	Eco-intensity of waste kg/USD
	7	Eco-capacity of air (total amount of pollution per capita), kg
	8	Eco-capacity of water resources (total amount of wastewater per capita), m^3^
	9	Eco-capacity of waste (total amount of waste per capita), t

**Table 4:** Aggregated and composite indicators of the Republic of Buryatia from 2010–2018.

	Indicators	2010	2011	2012	2013	2014	2015	2016	2017	2018
1	$I_{econ}$	0.2219	0.2272	0.1923	0.1759	0.1333	0.0957	0.0794	0.1328	0.1483
2	$I_{soc}$	0.4041	0.4396	0.4739	0.5038	0.4907	0.3797	0.3785	0.3994	0.3988
3	$I_{env}$	0.8970	0.9080	0.9072	0.8928	0.8904	0.8554	0.8630	0.8644	0.8635
4	$I_{reg}$	0.4317	0.4493	0.4357	0.4293	0.3876	0.3144	0.2960	0.3579	0.3710

## Data Availability

No new data were created or analyzed in this study. Data sharing is not applicable to this article.
